# Gendered Patterns of Engagement With NHS Health Checks in a Super‐Diverse City: Community Perspectives and Implications for Equity

**DOI:** 10.1002/puh2.70331

**Published:** 2026-08-05

**Authors:** Muhammad Hossain

**Affiliations:** ^1^ Public Health, Department of Life and Sports Sciences, School of Life and Health Sciences Birmingham City University City South Campus Birmingham UK

**Keywords:** community engagement, ethnic minority communities, gender differences, health inequalities, NHS Health Checks, preventive healthcare

## Abstract

**Background:**

NHS Health Checks are a national preventive programme in England designed to reduce cardiovascular disease and related long‐term conditions, yet uptake remains uneven. Gender differences persist, with men, particularly from racially marginalised and socioeconomically deprived communities, engaging less with preventive services, potentially reinforcing health inequalities.

**Methods:**

A qualitative community‐based participatory study was conducted in Birmingham using focus groups across 10 community organisations, alongside discussions with NHS Health Check professionals. Data were analysed using framework analysis, with attention to gendered and intersectional influences on engagement.

**Results:**

Women generally reported higher participation than men, although patterns varied across communities. Men's lower engagement was commonly attributed to inflexible employment, perceived low relevance of preventive care and limited trust in services. Women's engagement was shaped by childcare responsibilities, gender concordance of staff and cultural acceptability. Across genders, participants highlighted communication gaps, limited follow‐up and structural barriers within service delivery.

**Conclusion:**

Gendered inequalities in NHS Health Check engagement reflect intersecting social, cultural and systemic factors. Addressing these requires gender‐responsive, culturally competent delivery models, flexible access arrangements and strengthened community partnerships to promote equitable uptake of preventive care. These findings inform policy, commissioning and practice aimed at reducing gendered inequalities in prevention services.

## 1 Introduction

NHS Health Checks constitute a flagship preventive programme in England, designed to identify vascular risk factors and reduce future burden of cardiovascular disease (CVD), Type 2 diabetes, stroke, kidney disease and (in older adults) dementia risk factors [1]. Despite the intended equity function, uptake is uneven across population subgroups, with systematic evidence indicating higher attendance among women and older adults, and variability by deprivation and ethnicity [2, 3]. These patterns matter because gendered differences in preventive service engagement can amplify avoidable morbidity and premature mortality, particularly in urban settings where deprivation and ethnic diversity intersect.

Gendered engagement with preventive healthcare is shaped not only by individual attitudes, but also by wider social norms, divisions of labour and structural constraints [4]. Research suggests that men may be less likely to engage with preventive services where such engagement is perceived as low priority or inconsistent with norms of stoicism and self‐reliance, whereas women's participation may be constrained by caring responsibilities and the gendered organisation of everyday life [5, 6].

Birmingham provides a salient case: The city is highly diverse and experiences high levels of deprivation and associated health inequalities [7]. The Birmingham ‘NHS Health Check Focus Groups’ project engaged 193 participants (including 13 NHS Health Check professionals) from 10 ‘global majority’ groups and a White British group, offering an opportunity to examine how gendered engagement is experienced and understood within communities [8, 9]. The project data indicate higher participation among women than men, with particularly low participation among eligible Arab and White British men in the focus group sample. This manuscript analyses participant and professional accounts of the gendered factors shaping these patterns, including work and time scarcity, cultural norms, help‐seeking practices and service design, and develops actionable recommendations for commissioners and providers.

## 2 Background

A consistent finding in NHS Health Check evaluation and synthesis literature is that women are more likely to attend than men [2, 8, 9]. Existing evidence suggests several possible contributors to gendered patterns of engagement, including women's greater routine contact with primary care, gendered norms around help‐seeking and prevention, and differential responses to invitation and reminder systems [10]. Invitation method also shapes uptake; personalised prompts and outreach can improve attendance and may have equity effects [11]. Gender, however, does not operate in isolation: It interacts with ethnicity, migration, language, religion and deprivation [4]. Birmingham's report explicitly highlights how practical constraints (e.g., inflexible appointment times, transport and childcare) and cultural/linguistic barriers can vary by group and may differentially affect men and women.

Evidence also suggests that mistrust and negative prior experiences in primary care can undermine engagement among ethnic minority communities, including the extent to which patients feel listened to and respected [12]. Such dynamics can be gendered: Men may be more likely to avoid preventive care where there is limited perceived benefit or where attendance conflicts with work and provider roles; women may face childcare and domestic responsibilities alongside constraints in gender‐concordant care in some faith contexts. Accordingly, improving uptake requires intervention at multiple levels: communication, access model, cultural competence and follow‐up pathways. Community outreach models and telephone outreach by culturally concordant workers have shown promise for improving engagement among deprived and minority communities and are plausibly relevant for male engagement in hard‐to‐reach groups [13].

Previous research has documented inequalities in NHS Health Check uptake and broader barriers to preventive service engagement, including invitation methods, patient characteristics and contextual constraints [2, 14]. However, less is known about how gendered barriers are experienced and negotiated within super‐diverse urban communities where ethnicity, migration, work, caregiving, trust and communication intersect. The contribution of this short communication, therefore, lies not in claiming that gendered barriers are new but in providing context‐specific insight into how participants and practitioners understood the role of work patterns, childcare responsibilities, cultural expectations, trust and communication in shaping engagement with NHS Health Checks in Birmingham.

## 3 Aim

To examine gendered barriers and facilitators influencing engagement with NHS Health Checks among diverse communities in Birmingham and to identify service adaptations to reduce inequalities.

### 3.1 Objectives

1. Describe gendered patterns of NHS Health Check attendance within the focus group sample and explore perceived reasons.
2. Identify barriers and facilitators that are experienced as gendered (e.g., employment/time scarcity, childcare, cultural norms and staff gender preferences).
3. Develop gender‐responsive recommendations that are feasible within local commissioning and delivery constraints.

### 3.2 Research Questions

1. How do men and women describe their awareness, motivations and experiences of NHS Health Checks across communities?
2. Which barriers are perceived as particularly salient for men and which for women?
3. What delivery and communication adaptations are acceptable to communities and consistent with equity aims?

## 4 Methodology

### 4.1 Design

This short communication draws on qualitative focus group data generated through a wider Birmingham City Council–Birmingham City University community partnership on NHS Health Checks. The study was nested within a co‐produced participatory research framework, in which community organisations supported recruitment, language access, facilitation and contextual interpretation. The wider project explored barriers and facilitators to NHS Health Check engagement among diverse communities in Birmingham; the present article reports a focused gender analysis of patterns of engagement, access barriers and service implications.

### 4.2 Sample and Setting

The wider project comprised 22 focus group discussions: 20 with community participants and 2 with NHS Health Check professionals. Community participants were recruited through community organisations, faith settings and local networks. Focus groups were conducted in community settings and, where needed, facilitated in participants’ preferred languages with support from trusted community facilitators. Community participants represented 10 ethnic groups: Arab, Bangladeshi, Black Caribbean, Chinese, Ghanaian, Indian, Nigerian, Pakistani, Somali and White British communities. The community sample included 180 participants, of whom 83 were men and 97 were women. The mean age of community participants was 52.1 years; 153 were within the NHS Health Check eligible age range of 40–74 years, and 27 were approaching eligibility. Forty‐five community participants reported previous NHS Health Check attendance. The professional sample included 13 NHS Health Check professionals: nine women and four men, aged 30–50 years, including GPs, nurses and practice managers from Black/African and British Asian backgrounds. The wider project also included nine one‐to‐one interviews, but these were outside the scope of this short communication and were not included in the present analysis.

### 4.3 Data Collection

Semi‐structured topic guides explored awareness and understanding of NHS Health Checks, previous experiences, barriers and facilitators, the customer journey from invitation to follow‐up, appointment access, cultural relevance, communication preferences and suggestions for service improvement. Focus groups with NHS Health Check professionals additionally explored operational constraints, delivery processes, patient engagement, language support, cultural competence and follow‐up pathways. Discussions were audio‐recorded where appropriate and with consent, supplemented by field notes and recorded using standardised focus group reporting templates. Focus group providers received training and support through standardised workshops before data collection.

### 4.4 Data Analysis

Data were analysed using framework analysis, which is appropriate for applied qualitative health research where analysis needs to remain both systematic and policy‐relevant [15, 16, 17]. The analytic framework was informed by the study aims and topic guide domains, including awareness, previous experience, customer journey, cultural relevance, communication, access and service improvement. Within this framework, inductive coding was used to identify themes emerging from participants’ accounts, with particular attention to how gender intersected with work, caring responsibilities, cultural acceptability, communication, trust and follow‐up. Initial coding and interpretation were undertaken by the lead author and discussed with members of the wider research team to refine the thematic interpretation. NHS Health Check professionals’ accounts were used to contextualise community participants’ accounts, rather than to generate a separate professional‐facing analysis within this short communication. Reporting was informed by the consolidated criteria for reporting qualitative research (COREQ), which provides guidance for transparent reporting of qualitative interviews and focus groups [18].

In interpreting the findings, the descriptive attendance data were used only to contextualise the qualitative analysis. Although women in the sample reported slightly higher previous NHS Health Check attendance than men, the study was not designed to estimate population‐level gender differences in uptake. The findings are therefore presented as participant and professional accounts of possible explanations for gendered engagement, rather than as causal explanations.

### 4.5 Findings

The findings below should be read alongside Table 1, which provides descriptive context for the gender composition of the sample and self‐reported previous NHS Health Check attendance. The qualitative findings are presented as participant and professional accounts of possible explanations for gendered engagement, rather than as causal explanations of uptake.

**TABLE 1:** Community participant characteristics and self‐reported NHS Health Check attendance by ethnic group and gender.

Ethnic group	Men (*n*)	Women (*n*)	Total (*n*)	NHS Health Check eligible (*n*)	Reported attendance among men (*n*)	Reported attendance among women (*n*)	Total reported attendance (*n*)
Arab	7	10	17	15	0	1	1
Bangladeshi	9	12	21	18	2	2	4
Black Caribbean	9	9	18	15	1	2	3
Chinese	7	9	16	15	3	3	6
Ghanaian	8	6	14	8	3	4	7
Indian	8	12	20	20	1	1	2
Nigerian	7	13	20	17	4	4	8
Pakistani	12	12	24	20	3	5	8
Somali	10	10	20	17	2	2	4
White British	6	4	10	8	0	2	2
Total	**83**	**97**	**180**	**153**	**19**	**26**	**45**

*Note:* Eligibility refers to community participants aged 40–74 years. Reported attendance refers to self‐reported previous NHS Health Check attendance among community participants. These figures are descriptive and should not be interpreted as representative NHS Health Check uptake rates for Birmingham. NHS Health Check professionals were included for service‐delivery perspectives and are not included in eligibility or attendance calculations.

#### 4.5.1 Gendered Attendance Patterns: Women's Higher Reported Engagement and ‘Missing Men’

The descriptive data provided context for the qualitative analysis. Among the 180 community participants, 97 were women, and 83 were men. Forty‐five participants reported previous NHS Health Check attendance, comprising 26 women and 19 men. This indicates slightly higher reported engagement among women in this sample, although the pattern was uneven across ethnic groups and should not be interpreted as a population‐level estimate of uptake. Reported attendance was particularly low among some male groups, including Arab and White British men in this sample, suggesting potential priority groups for targeted outreach.

Participants’ accounts suggested several possible explanations for lower male engagement, including paid work pressures, low perceived need for preventive care and the tendency to prioritise health services only when symptoms are present. Some participants described preventive checks as less relevant when they felt healthy, for example: ‘*I'm young and healthy, so I don't think I need it’*. This type of account does not establish why men attended less, but it suggests that perceptions of risk, health responsibility and the value of prevention may shape how some participants understood NHS Health Checks.

#### 4.5.2 Work, Time Scarcity and the Economics of Attendance

Work and time scarcity emerged as important gendered dimensions of engagement. Men's accounts, and some women's accounts about male relatives, often described inflexible working patterns, long hours, income loss and difficulty attending appointments during standard working times. As one participant explained, ‘*I work such long hours at my shop that I simply couldn't take time off to go to the GP for a check‐up’*. Another participant similarly noted, ‘*Appointments are only during work hours, which makes it impossible for me to attend without taking time off’*.

Women also described time‐related barriers, but these were more commonly connected to unpaid caring responsibilities, childcare, family organisation and the timing of appointments. One participant stated, ‘*I look after my children all day, so I can't go to an appointment because I don't find childcare at short notice’*. These accounts suggest that time scarcity was not experienced in a uniform way. For some men, attendance was framed around paid work and potential income loss, whereas for some women it was shaped by caring responsibilities and the practical organisation of family life. In both cases, the issue was not simply individual motivation, but the fit between NHS Health Check delivery and participants’ everyday responsibilities.

#### 4.5.3 Gendered Cultural Norms and Acceptability of Delivery

Participants across several communities described the acceptability of NHS Health Checks as shaped by gender, cultural expectations, modesty, language and trust in staff. Women in particular discussed the importance of feeling comfortable with the person delivering the check, especially where discussions involved sensitive health issues. As one participant stated, ‘*I would feel more comfortable with a female doctor who understands my history*’.

This finding suggests that gender‐concordant and culturally sensitive delivery may influence whether some women perceive NHS Health Checks as accessible and acceptable. However, women's experiences should not be reduced to preference for female staff alone. Their accounts also highlighted the importance of appointment timing, language, familiarity with services, trust in professionals and recognition of family responsibilities. For men, cultural norms were discussed more indirectly, particularly in relation to reluctance to seek preventive care, concerns about appearing vulnerable, and the tendency to engage with health services only when unwell. These findings suggest that gendered acceptability operates through multiple intersecting factors rather than through a single cultural explanation.

#### 4.5.4 Communication Channels and Trust

Communication and trust emerged as cross‐cutting influences on engagement rather than purely gender‐specific barriers. Participants and professionals both described standard invitation letters as insufficient for some communities, particularly where English language barriers, low awareness or mistrust of formal systems were present. One participant explained, ‘*In our community, people are more likely to trust information coming from an authoritative figure such as a leader. A letter does not have the same effect*’. Another participant stated, ‘*If someone from the mosque or a community group explains it to us, we will listen and take it seriously’*.

These accounts suggest that trusted community channels may improve the credibility and perceived relevance of NHS Health Check invitations. The gender relevance of this theme lies in how communication pathways may reach men and women differently. Men who are less likely to respond to passive invitations may require more direct, trusted or workplace‐sensitive outreach, whereas women may benefit from community‐based communication that recognises caring roles, language needs and culturally acceptable delivery. NHS Health Check professionals’ accounts broadly aligned with community participants’ accounts, particularly in relation to language barriers, low awareness, trust and the need for clearer explanations of the purpose and benefits of the check.

#### 4.5.5 Follow‐Up as a ‘Value Signal’

Follow‐up was described as an important part of how participants judged the value and seriousness of NHS Health Checks. Participants and professionals suggested that when follow‐up was unclear, delayed or absent, the check could be perceived as a one‐off procedure rather than part of a meaningful preventive care pathway. One participant reported, ‘*I had to call the GP several times to get my results. It felt like they didn't care’*. Another participant described uncertainty after being advised that further tests were needed: ‘*They told me I needed more tests, but they didn't explain why or what would happen next’*.

These accounts suggest that follow‐up functioned not only as a clinical process, but also as a relational signal that the participant's attendance mattered. This theme was not gender‐specific in a simple sense, but it has implications for gendered engagement. Where men already perceive low benefit relative to the time or income costs of attending, weak follow‐up may further reduce the perceived value of future engagement. For women managing caring responsibilities and competing demands, clear follow‐up may help justify the time and effort required to attend. Professional participants also emphasised that health checks need to be linked to clear advice, onwards referral and practical support if they are to be seen as worthwhile by communities.

## 5 Discussion

This study adds to existing literature showing that NHS Health Check uptake is patterned by social and demographic inequalities, including gender, by offering context‐specific insight into factors that participants and practitioners perceived as shaping engagement. These included work and time scarcity, caring responsibilities, low perceived need for preventive care, communication barriers, trust, cultural expectations, language and deprivation [2, 3, 10]. The Birmingham data suggest that gendered engagement with NHS Health Checks is not shaped by a single barrier, but by the cumulative effect of barriers operating across the service pathway, from invitation and booking to delivery, advice and follow‐up.

Outreach and the invitation methods are therefore particularly important [19]. Evidence from previous research suggests that the design of invitations and follow‐up interventions can influence uptake, including among communities experiencing socioeconomic and ethnic inequalities. For example, culturally concordant telephone follow‐up has been shown to support engagement by reducing communication barriers and increasing the perceived relevance of the invitation [13]. In the present study, participants’ emphasis on trusted local channels and the need to counter misinformation suggests that ‘who delivers the message’ may be as important as ‘what the message says’. This is particularly relevant for participants who were uncertain about formal letters, mistrustful of unfamiliar communication or unsure whether NHS Health Checks were relevant when they felt well.

The findings also show that gendered engagement needs to be understood intersectionally. Men's lower perceived need for preventive care, work‐related time constraints and reluctance to seek help interacted with wider factors such as language, cultural expectations, deprivation and trust. Women's engagement was shaped by caring responsibilities, appointment timing, privacy, gender‐sensitive provision and confidence that the service would offer meaningful follow‐up. These patterns suggest that improving uptake requires more than additional invitations; it requires gender‐responsive and culturally credible pathways that make NHS Health Checks visible, understandable, practically accessible and worthwhile after attendance.

## 6 Limitations

This study has several limitations. First, participants were recruited through community organisations, faith settings and local networks, meaning the sample may over‐represent individuals already connected to community groups and under‐represent more socially isolated residents, those with limited trust in statutory services, or those least engaged with preventive healthcare. Although the study included participants from 10 community groups, some subgroup numbers were small, and the study was not designed to rank ethnic groups, estimate population‐level uptake or identify definitive priority populations. References to specific ethnic or community groups should therefore be interpreted cautiously. Instead, these references are used to illustrate how gender, language, culture, migration history, work patterns, caring responsibilities and trust may interact in shaping engagement with NHS Health Checks in this sample. Second, as a qualitative short communication based on focus group data, the findings are not statistically generalisable beyond Birmingham, although they may offer transferable insights for other diverse urban settings. Third, some accounts reflected participants’ perceptions of wider community attitudes, including men's engagement with NHS Health Checks, rather than only their own direct experiences. Fourth, previous NHS Health Check attendance was self‐reported, and gender‐specific eligibility data were not available in sufficient detail to calculate precise uptake rates among eligible men and women. Finally, although the analysis identifies possible explanations for gendered engagement, it cannot establish causal relationships between gender, service design and NHS Health Check attendance.

## 7 Conclusions

This study suggests that gendered engagement with NHS Health Checks in Birmingham was shaped by intersecting barriers related to work, caring responsibilities, perceived need, communication, culture, trust and deprivation. These findings should be interpreted as participant and practitioner accounts rather than population‐level estimates of uptake. To reduce inequalities, NHS Health Check delivery should move beyond standard invitation letters towards gender‐responsive, culturally competent and community‐informed approaches. This includes proactive outreach, clearer communication, flexible appointment options, community‐based delivery, gender‐sensitive provision where appropriate, and stronger follow‐up after risk has been identified. Without such changes, NHS Health Checks may continue to miss some of the groups who could benefit most from preventive support. With these changes, the programme has greater potential to support equitable CVD prevention in super‐diverse cities.

## Author Contributions

**Muhammad Hossain**: conceptualisation, investigation, funding acquisition, writing – original draft, methodology, validation, visualisation, writing – review and editing, software, formal analysis, project administration, data curation, supervision, resources.

## Funding

This study was supported by Birmingham City Council as part of the NHS Health Checks community engagement project.

## Disclosure

The views expressed in this manuscript are those of the author and do not necessarily represent the views of Birmingham City Council.

## Ethics Statement

Ethical approval for this study was granted by the Birmingham City University Health, Education and Life Sciences Faculty Academic Ethics Committee. The ethics approval reference was Hossain/#13344/sub2/R(A)/2024/Sep/HELS FAEC.

## Consent

All participants received an information sheet outlining the study purpose, voluntary nature of participation, confidentiality, data handling and right to withdraw. Written informed consent was obtained from all participants prior to participation, including consent for audio recording where applicable and for the use of anonymised quotations in reports, publications and dissemination outputs. No identifiable personal information is reported in this manuscript.

## Conflicts of Interest

The author declares no conflicts of interest.

## Data Availability

The data that support the findings of this study are available from the corresponding author upon reasonable request.
